# Trends and inequalities in stunting in Nepal: a secondary data analysis of four Nepal demographic health surveys from 2001 to 2016

**DOI:** 10.1186/s40795-019-0283-x

**Published:** 2019-03-04

**Authors:** Sajama Nepali, Padam Simkhada, Ian Davies

**Affiliations:** 1 Central Department of Home Science, Tribhuvan University, Kathmandu, Nepal; 2 Faculty of Education, Health and Community, Public Health Institute, Liverpool John Moores University, Liverpool, UK; 3 Faculty of Education Health and Community, Sport Studies, Leisure and Nutrition, Liverpool John Moores University, Liverpool, UK

**Keywords:** Children, Inequalities, Socio-demographic, Stunting, Trends, Wealth quintiles

## Abstract

**Background:**

The rate of stunting in Nepal is among the highest in the world, which is a major public health problem. The objective of this study was to present data on stunting prevalence according to socio-demographic and geographical circumstances and to determine the impact of those circumstances on the risk of stunting.

**Methods:**

Data from Nepal Demographic and Health Surveys were used with the study population of children under 5 years old. The prevalence of stunting was determined by descriptive analysis and logistic regression analysis was used to determine risk factors for stunting.

**Results:**

The prevalence of stunting has declined in overall as well as in all groups and subgroups analysed. The percentage of stunted children from 2001 to 2016 decreased by 18 and 10.7% in the rural and urban areas respectively. The unadjusted analysis depicted association between stunting and children living in rural areas since children living in rural areas had higher odds of being stunted compared to their urban counterparts. However, the association was no longer observed when adjusted for other variables included in this study. Children born to mothers without any education had 2.27 (95% CI 1.70–3.05), 5.222 (95% CI 2.54–10.74), 1.81 (95% CI 0.92–3.55) and 1.92 (95% CI 1.28–2.89) odds of being stunted than those born to mothers with higher education for the year 2001, 2006, 2011 and 2016 respectively in the adjusted analysis. Similarly, children belonging to the poorest wealth quintile had 1.90 (95% CI 1.55–2.33), 1.87 (95% CI 1.36–2.58), 2.47 (95% CI 1.51–4.02) and 4.18 (95% CI 2.60–6.71) odds of being stunted than those belonging to the richest quintile in 2001, 2006, 2011 and 2016 respectively. The association between stunting and wealth quintile depicting children belonging to the poorest and poorer wealth quintile having higher odds of being stunted remain the same in both unadjusted and adjusted analysis.

**Conclusion:**

At national level, stunting is decreasing in Nepal; however, the prevalence of stunting is different between groups and subgroups analysed. The substantial inequalities in stunting have been preserved. Therefore, special emphasis should be given to vulnerable groups such as children belonging to the poorest and poorer wealth quintile instead of using blanket approach for delivering nutrition interventions. A balanced approach to nutritional inequalities prevalent across different regions and subgroups is required.

## Background

Linear growth of children during early life correlates with long term health and productivity and, as it is partly a reflection of the environment in which they are growing up, may be considered as an indicator of a country’s overall development [1]. Stunting (the percentage of children aged 0 to 59 months, whose height for age is below − 2.00 to − 2.99 standard deviation (SD) for moderate and − 3.00 SD for severe stunting from the median of the 2006 World Health Organization (WHO) Child Growth Standards) occurs during the first thousand days of life, starting from conception to the second birthday [2]. The established conceptual framework for considering causes of stunting and other forms of malnutrition considers proximal causes such as insufficient nutrient intake, frequent infections and other diseases, and more distal basic causes such as socio-economic, political, cultural and socio-demographic factors, which this study focuses on.

Worldwide, 154.8 (22.9%) million children under 5 years were reported stunted in 2016 [3]. There are 86.5 (23.9%) million stunted children under 5 years living in Asia, of which 61.9 million (35.8%) belongs to South Asia [3]. The prevalence of stunting has been decreasing in Nepal from 57.2% in 2001 [4] to 35.8% in 2016 [5]. Nepal suffered 10 years of armed conflict period that came to an end in 2006, building a peace agreement between the Maoists and the Government and is now in a transition phase to peace and stability after several years of political instability [6]. In Nepal, 51% of households are suffering from food insecurity and do not have adequate access to food throughout the year [7]. This is highest in the mountainous region of the country (13.8%), compared to the hill (10.0%) and terai (9.2%) regions. [5]. In addition, nearly all households belonging to the bottom wealth quintile are victims of food insecurity [7]. Food insecurity positively correlates with stunting, as the threat of stunting increases with the level of food insecurity [8].

While the average percentage of stunted children has reduced, the prevalence is different between various socio-demographics and economic subgroups. In order to strengthen interventions to combat the levels of child undernutrition and reduce the stunting prevalence, it is crucial to separate the prevalence and trends of stunting according to socio-demographics and economic subgroups. In response to this need in Nepal, the present study presents the trends of stunting prevalence among children under 5 years old for the subsequent survey years.

## Methods

Nepal Demographic and Health Survey (NDHS) is a nationally representative cross sectional survey conducted every 5 years [9]. The surveys employed two stage stratified cluster sampling [4, 5, 10, 11]. The detail of sampling is provided in the freely available NDHS reports [4, 5, 10, 11]. The study population was children under 5 years old and thus child dataset was used. The total sample size analysed in this study was 16,606 for the four surveys, with a response rate of 96.1%, 96%, 95.3% and 95.9% for the year 2001, 2006, 2011 and 2016 respectively.

### Variables

The dependent variable was stunting. The main explanatory independent variable was wealth quintile, which was recorded as (i) poorest (ii) poorer (iii) medium (iv) richer and (v) richest. The wealth quintile is developed using the statistical procedure known as principal component analysis, that categorized the above into five categories based on household’s ownership of selected assets, such as televisions and bicycles; materials used for housing construction; and types of water access and sanitation. [4, 5, 10–12]. Other independent variables were place of residence, categorized into (i) rural and (ii) urban; mother’s education level, categorized into (i) no education (ii) primary (iii) secondary and (iv) higher; development region, categorized into (i) eastern (ii) central (iii) western (iv) mid-western and (v) far-western; and ecological region, categorized into (i) mountainous (ii) hill and (iii) terai (plain region) as per the altitude of the region.

### Statistical analysis

Data analysis was carried out using Statistical Package for Social Science (SPSS) version 23 (IBM USA) using descriptive analysis. Confidence intervals (CI) for the prevalence estimates were computed to identify statistical difference in prevalence of stunting between survey years. Similarly, logistic regression was carried out to identify the odds of being stunted with 95% CI after adjusting for all the five independent variables; residence, mother’s education, wealth quintile, development and ecological region. A *p* value < 0.05 was considered statistical significant for the association between stunting and independent variables. The DHS sampling design includes both under and over-sampling, hence, all analyses were conducted with sample-weighted data. Complex sample analysis method was used to account for the study design and sample weight [13].

### Ethics

All the NDHSs were approved by the ethics committee of Nepal Health Research Council and human research ethics committee in ICF Macro International [4, 5, 10, 11]. The detail of sampling is provided in the freely available NDHS reports [4, 5, 10, 11]. Similarly, Independent Review Boards of New Era and ICF Macro International reviewed and approved all the data collection tools and procedures for NDHSs. The data was made publicly available after removing personal identifiers to render it anonymous. Therefore, it was accessed through the DHS program website upon request and submission of proposal noting the use of the dataset [14].

## Results

Stunting trends by residence, mother’s education, wealth quintile, development and ecological regions.

**Fig. 1:**
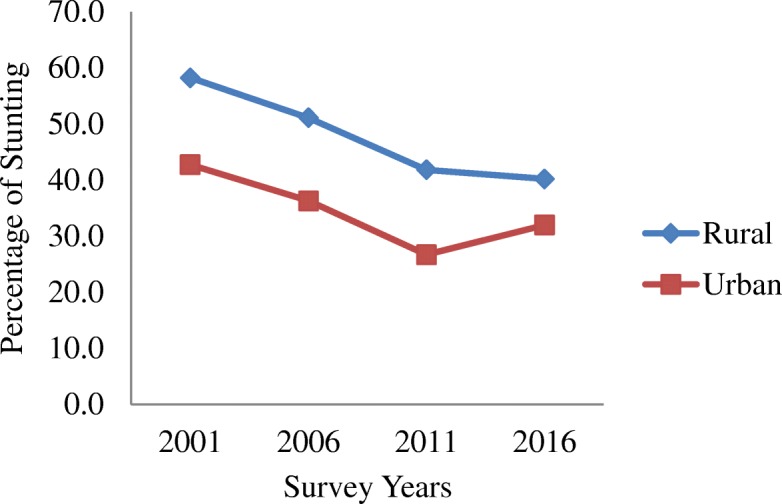
Trend of stunting among children under 5 years according to place of residence for the year 2001, 2006, 2011 and 2016

**Fig. 2:**
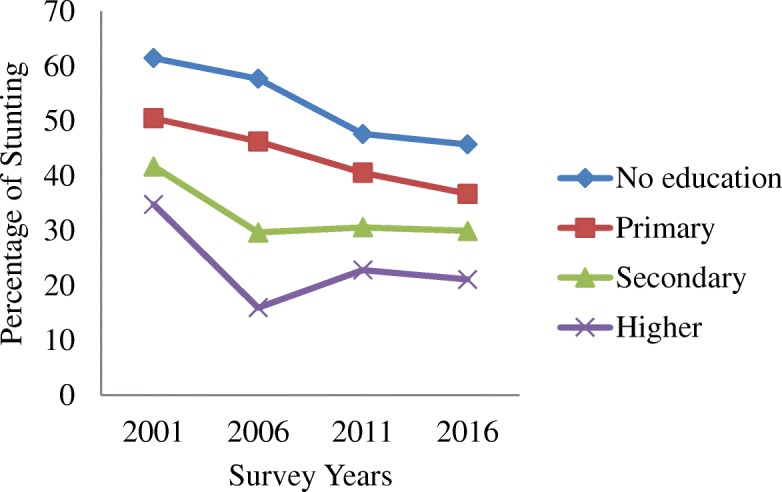
Trend of stunting among children under 5 years according to mother’s education for the year 2001, 2006, 2011 and 2016

**Fig. 3:**
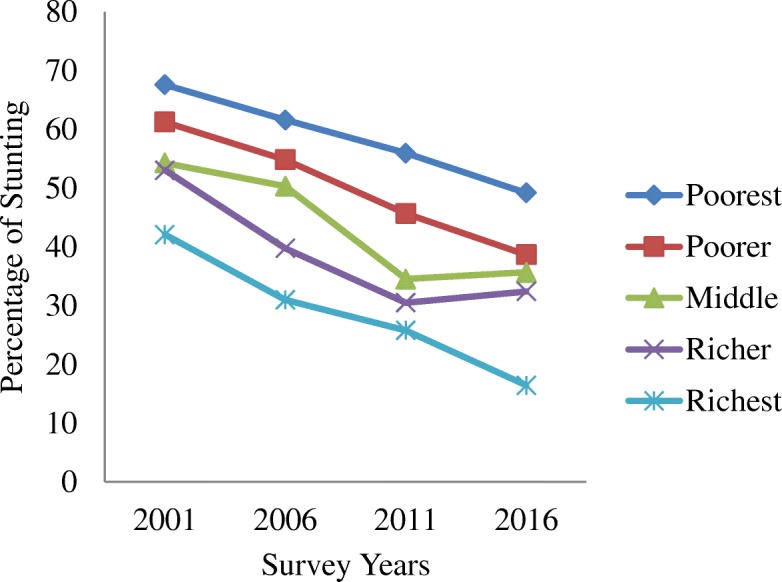
Trend of stunting among children under 5 years according to wealth quintiles for the year 2001, 2006, 2011 and 2016

**Fig. 4:**
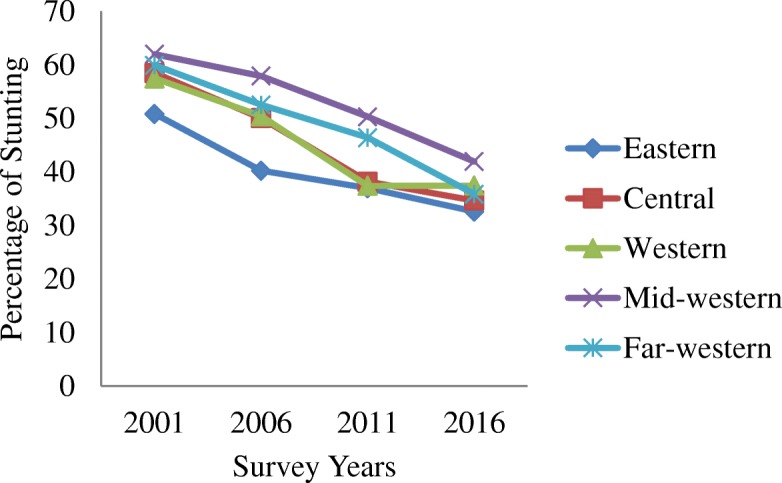
Trend of stunting among children under 5 years according to development region for the year 2001, 2006, 2011 and 2016

**Fig. 5:**
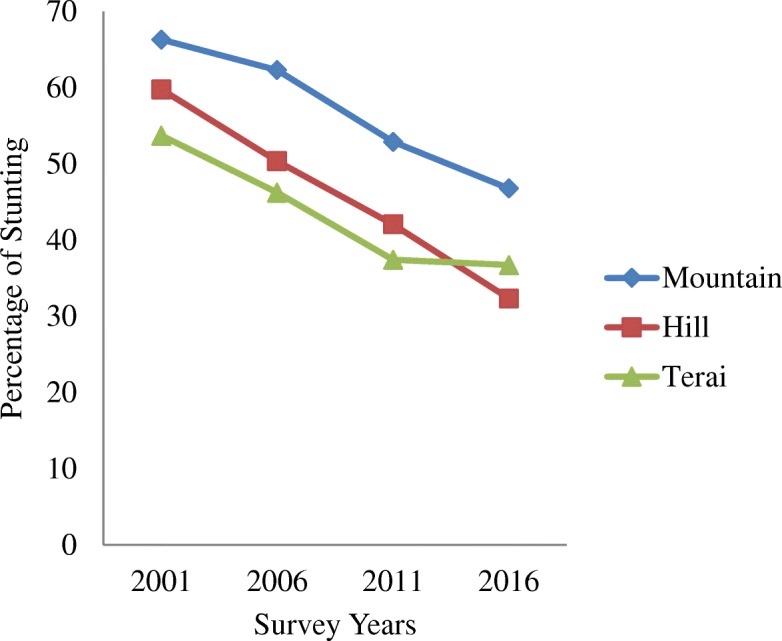
Trend of stunting among children under 5 years according to ecological region for the year 2001, 2006, 2011 and 2016

Table 1 shows the trends in stunting by residence, region, mother’s education and socio-economic status from 2001 to 2016. The average prevalence of stunting has decreased from 51% in 2001 to 35.8% in 2016. Similarly, it has also decreased among the groups and subgroups analysed as illustrated in Figs. 1, 2, 3, 4 and 5. The percentage of stunted children decreased by 18 and by 10.7% in the rural and urban areas respectively in the span of 15 years (Fig. 1). Although the prevalence of stunting declined in both the rural and the urban areas, a clear inequality gap in the stunting levels was observed. Among mothers with no education, the prevalence of stunting in children under 5 years decreased from 61.4% in 2001 to 45.7% in 2016. In 2001, there was a gap of 26.7% between mothers with no education and mothers with higher education and in 2006, it widened up to 41.7% and then decreased to 24.6% in 2016 (Fig. 2). The prevalence of stunting decreased by 18.4% from 2001 to 2016 for the poorest quintile and for the richest quintile, the reduction was by 25.6% as shown in Fig. 3. The inequality has expanded over 15 years. The prevalence of stunting for children from the poorest wealth quintile was twice or more to that of children from the richest quintile for year 2006, 2011 and 2016. The mid-western development region experiences the highest prevalence of stunting among all the regions and across the four survey years (Fig. 4). Comparing the mid-western with the eastern region, the gap in stunting between these two regions has increased by 1.8% in 15 years. Similarly, considering ecological region, the prevalence of stunting was highest in the mountainous region in all four survey years (Fig. 5). Comparing the data of 2016 with 2011, the decline has been gradual for the mountainous (6.1%) and the hill region (9.8%) except for the terai region, where the decline is less than 1 % (0.7%). In addition to this, the prevalence in the mountainous region exceeded the national average across the 4 years.

**Table 1:** Stunting trend by residence, mother’s education, wealth quintile, development and ecological regions

Characteristics	2001		95%CI	2006		95%CI	2011		95%CI	2016		95%CI
	n	%	(Lower-Upper)	n	%	(Lower-Upper)	n	%	(Lower-Upper)	n	%	(Lower-Upper)
Type of place of residence												
Rural	3503	58.23	57.02–59.43	2362	51.10	49.74–52.45	948	41.82	39.88–43.76	459	40.19	38.24–42.15
Urban	182	42.74	41.53–43.94	231	36.30	35.00–37.60	58	26.69	24.95–28.43	409	31.98	30.12–33.83
Mother’s education												
No education	2836	61.46	60.27–62.65	1771	57.72	56.39–59.06	549	47.64	45.67–49.60	378	45.72	43.74–47.71
Primary	452	50.50	49.28–51.72	426	46.27	44.92–47.62	195	40.56	38.63–42.49	174	36.74	34.82–38.66
Secondary	224	41.74	40.54–42.95	294	29.68	28.44–30.91	203	30.63	28.82–32.44	225	29.98	28.16–31.81
Higher	78	34.80	33.63–35.96	19	15.98	14.99–16.97	29	22.83	21.18–24.48	67	21.10	19.48–22.73
Wealth quintiles												
Poorest	1096	67.62	66.48–68.76	812	61.60	60.28–62.91	549	55.97	54.01–57.92	244	49.21	47.22–51.20
Poorer	863	61.26	60.07–62.45	614	54.85	53.51–56.20	195	45.70	43.74–47.66	204	38.69	36.75–40.63
Middle	700	54.29	53.07–55.51	541	50.35	49.00–51.70	203	34.54	32.67–36.41	196	35.69	33.78–37.60
Richer	639	53.06	51.84–54.28	382	39.77	38.45–41.09	29	30.52	28.71–32.33	170	32.43	30.57–34.30
Richest	387	42.11	40.91–43.32	243	31.01	29.75–32.26	86	25.80	24.08–27.52	53	16.47	14.99–17.95
Development region												
Eastern	759	50.84	49.62–52.06	467	40.25	38.92–41.57	221	36.97	35.07–38.87	177	32.59	30.73–34.46
Central	1233	58.47	57.27–59.68	854	50.05	48.7–51.4.00	294	38.20	36.29–40.11	301	34.68	32.78–36.57
Western	690	57.49	56.28–58.70	492	50.43	49.08–51.78	174	37.40	35.49–39.30	174	37.46	35.53–39.39
Mid-western	603	62.00	60.82–63.19	383	57.93	56.59–59.26	188	50.32	48.36–52.29	140	41.96	39.99–43.92
Far-western	399	59.93	58.73–61.12	397	52.54	51.20–53.89	129	46.42	44.46–48.38	76	35.87	33.96–37.78
Ecological zone												
Mountain	324	66.31	65.16–67.47	279	62.31	61.00–63.62	104	52.90	50.94–54.86	80	46.79	44.81–48.78
Hill	1609	59.77	58.57–60.97	1089	50.37	49.02–51.72	417	42.08	40.14–44.02	283	32.34	30.47–34.20
Terai	1752	53.72	52.50–54.93	1225	46.24	44.90–47.59	485	37.42	35.51–39.32	505	36.73	34.81–38.65

“n” referred to frequency of “stunted children” under 5 years only. Children who slept in the household the night before the survey and who have complete information on date of birth were selected for analysis for the year 2001, 2006 and 2011. For 2016, children who slept in the household the night before the survey only were selected. Data weighted according to DHS recommendations [13]

Association between stunting and residence, region, mother’s education and socio-economic status.

Table 2 shows the crude odds ratio (COR) and adjusted odds ratio (AOR) with 95% CI for stunting and its relationship with residence, region, mother’s education and socio-economic status. Children living in the rural areas were associated with increased odds of stunting compared to their urban counterparts in the crude analysis; however, no association was seen in the adjusted analysis for all the survey years. Children born to mothers without any education had 2.27 (95%CI 1.70–3.05) odds of being stunted than those born to mothers with higher education in 2001. This odds increased to 5.22 (95%CI 2.54–10.74) in 2006, then decreased to 1.81 (95%CI 0.92–3.55) in 2011 and again increased to 1.92 (95%CI 1.28–2.89) in 2016. Children belonging to the poorest wealth quintile had 1.90 (95%CI 1.55–2.33) odds of being stunted than those belonging to the richest quintile in 2001. The odds dropped to 1.87 (95%CI 1.36–2.58) in 2006 and then increased to 2.47 (95%CI 1.51–4.02) and 4.18 (95%CI 2.60–6.71) in 2011 and 2016 respectively. While children under 5 years living in the mountain region experienced increased odds from 1.47 (95%CI 1.22–1.76) in 2001 to 1.55 (95%CI 1.16–2.08) in 2006, the odds decreased to 1.20 (95%CI 0.87–1.66) in 2011 and to 0.98 (95%CI 0.65–1.47) in 2016 compared to those living in terai region.

**Table 2:** Association between stunting and residence, region, mother’s education and socio-economic status

Characteristics	2001		2006		2011		2016	
	COR (95%CI)	AOR (95%CI)	COR (95%CI)	AOR (95%CI)	COR (95%CI)	AOR (95%CI)	COR (95%CI)	AOR (95%CI)
Type of place of residence	*P* < 0.001	*P* = 0.219	*P* < 0.001	*P* = 0.384	*P* < 0.001	*P* = 0.183	*P* = 0.001	*P* = 0.572
Rural	1.87 (1.58,2.2)	1.13 (0.93,1.36)	1.83 (1.52,2.2)	1.1 (0.89,1.37)	1.97 (1.53,2.55)	1.24 (0.9,1.69)	1.43 (1.16,1.77)	1.06 (0.86,1.32)
Urban	1	1	1	1	1	1	1	1
Mother’s education	*P* < 0.001	*P* < 0.001	*P* < 0.001	*P* < 0.001	*P* < 0.001	*P* = 0.116	*P* < 0.001	*P* < 0.001
No education	2.99 (2.26,3.95)	2.27 (1.7,3.05)	7.18 (3.75,13.73)	5.22 (2.54,10.74)	3.08 (1.74,5.43)	1.81 (0.92,3.55)	3.15 (2.22,4.46)	1.92 (1.28,2.89)
Primary	1.91 (1.37,2.67)	1.54 (1.09,2.17)	4.53 (2.35,8.74)	3.54 (1.73,7.23)	2.31 (1.25,4.27)	1.55 (0.77,3.12)	2.17 (1.51,3.12)	1.34 (0.91,1.98)
Secondary	1.34 (0.97,1.86)	1.23 (0.88,1.72)	2.22 (1.15,4.28)	1.99 (0.97,4.07)	1.49 (0.81,2.73)	1.27 (0.66,2.42)	1.6 (1.13,2.26)	1.25 (0.86,1.81)
Higher	1	1	1	1	1	1	1	1
Wealth Quintiles	*P* < 0.001	*P* < 0.001	*P* < 0.001	*P* < 0.001	*P* < 0.001	*P* < 0.001	*P* < 0.001	*P* = 0.083
Poorest	2.87 (2.37,3.48)	1.9 (1.55,2.33)	3.57 (2.84,4.49)	1.87 (1.36,2.58)	3.65 (2.58,5.18)	2.47 (1.51,4.02)	4.91 (3.39,7.12)	4.18 (2.6,6.71)
Poorer	2.17 (1.83,2.58)	1.43 (1.18,1.74)	2.7 (2.22,3.3)	1.67 (1.3,2.13)	2.42 (1.68,3.5)	1.7 (1.04,2.78)	3.2 (2.19,4.67)	2.6 (1.66,4.07)
Middle	1.63 (1.38,1.94)	1.14 (0.95,1.36)	2.26 (1.76,2.89)	1.54 (1.17,2.02)	1.52 (1,2.31)	1.15 (0.71,1.88)	2.81 (1.9,4.17)	1.99 (1.28,3.1)
Richer	1.55 (1.31,1.84)	1.16 (0.97,1.39)	1.47 (1.19,1.82)	1.15 (0.9,1.46)	1.26 (0.85,1.88)	1.05 (0.69,1.6)	2.43 (1.64,3.6)	1.82 (1.14,2.89)
Richest	1	1	1	1	1	1	1	1
Development regions	*P* < 0.001	*P* = 0.011	*P* < 0.001	*P* < 0.001	*P* = 0.009	*P* = 0.630	*P* = 0.214	*P* = 0.014
Central	1.36 (1.12,1.66)	1.33 (1.11,1.59)	1.49 (1.14,1.94)	1.51 (1.2,1.89)	1.05 (0.75,1.48)	0.99 (0.68,1.45)	1.1 (0.85,1.42)	1.11 (0.84,1.45)
Western	1.31 (1.03,1.67)	1.45 (1.14,1.83)	1.51 (1.17,1.95)	1.76 (1.38,2.25)	1.02 (0.7,1.47)	1.04 (0.7,1.54)	1.24 (0.87,1.77)	1.5 (1.06,2.11)
Mid-western	1.58 (1.28,1.94)	1.26 (1,1.57)	2.04 (1.59,2.63)	1.71 (1.33,2.19)	1.73 (1.18,2.54)	1.28 (0.87,1.87)	1.49 (1.06,2.1)	1.3 (0.91,1.85)
Far-western	1.45 (1.14,1.84)	1.13 (0.86,1.49)	1.64 (1.28,2.11)	1.26 (1.01,1.56)	1.48 (1.02,2.14)	1.11 (0.74,1.66)	1.16 (0.83,1.62)	0.91 (0.64,1.29)
Eastern	1	1	1	1	1	1	1	1
Ecological zones	*P* < 0.001	*P* < 0.001	*P* < 0.001	*P* = 0.012	*P* < 0.001	*P* = 0.167	*P* = 0.005	*P* = 0.003
Mountain	1.7 (1.42,2.03)	1.47 (1.22,1.76)	1.92 (1.5,2.47)	1.55 (1.16,2.08)	1.88 (1.4,2.52)	1.2 (0.87,1.66)	1.51 (1.07,2.15)	0.98 (0.65,1.47)
Hill	1.28 (1.08,1.51)	1.18 (1,1.38)	1.18 (0.96,1.44)	1.2 (0.98,1.45)	1.22 (0.96,1.55)	0.91 (0.7,1.2)	0.82 (0.66,1.03)	0.67 (0.5,0.89)
Terai	1	1	1	1	1	1	1	1

Children who slept in the household the night before the survey and who have complete information on date of birth were selected for analysis for the year 2001, 2006 and 2011. For 2016, children who slept in the household the night before the survey only were selected. Data weighted according to DHS recommendations [13]

## Discussion

The objective of this study was to present data on stunting prevalence according to socio-demographics and geographical circumstances from 2001 to 2016. When comparing the Nepal’s prevalence of stunting with India and Pakistan, whose per capita income are higher than Nepal, the prevalence of stunting in Nepal is lesser than both more developed South Asian countries by 12.2% for India in 2016 [15] and by 3.8% for Pakistan in 2012 [16]. On one hand, Nepal made impressive progress in reducing the prevalence of stunting with 21.4% reduction in 15 years, from 57.2% in 2001 to 35.8% in 2016. The reduction might be explained by upliftment in educational status of mothers, increase in access to health care, improvement in sanitation and implementation of integrated interventions [17–20]. The government doubled its investment in education from 10% in 1988/1992 to 20% in 2006/2011 leading to improvement in overall educational status including of mothers [17]. Aligning with increasing budget in education, several reformative activities were done in bringing about the positive changes in education such as implementation of Welcome to School Program that started in 2005 focusing on enrolment for girls and disadvantaged groups, and National Literacy Campaign launched in 2008 for both children and adult education [21]. The campaign was successful in lifting up the literacy of women aged 15–24 years close to the level of national average [21]. Alongside, the Comprehensive Peace Agreement made in November 2006 between the government and the Communist Party of Nepal-Maoist brought steadiness in operation of school activity that was missing during the period of armed conflict [21]. The provision of midday meals, separate toilets for girls and boys, free textbooks, residential schools for girls, and the presence of female teachers might have provided enabling environment for students, especially girls to continue going school [21]. Additionally, scholarships were provided to all girls student from 2010/11 onwards, which otherwise were only provided to 50% of enrolled girls [21]. The above mentioned interventions raised the literacy level of girls and women, who are apparently the future mothers. However, it was also noted that only 79.5% of the poorest quintile 15–24 years were literate compared to 98% from the richest quintile [21], which is similar to the result of this study. Similarly, the improvement in access to health sector was made by increasing budget in primary health care [19, 20] and by increasing the number of primary health care outreach clinics that provided grassroots health services, leading to significant improvements in immunizations, vitamin A supplementation, prenatal, neonatal and postnatal care (including nutritional advice), and treatment of common diseases, particularly diarrhoea, malaria, and acute respiratory infections [18]. Further to this, improvement in sanitation was also noted with notable reduction in open defecation, from 36 to 10% from 2011 to 2016 [5, 11]. The construction of low-cost toilet facilities through Community-Led Total Sanitation intervention not only decreased open defecation rates but also brought changes in behaviour of community people to follow hygienic sanitation practices [22]. Following the recommendation of Nepal Nutrition Assessment and Gap Analysis to roll out the nutrition specific and sensitive intervention, Nepal implemented a 5 year plan known as Multi-sectoral Nutrition Plan in 2012, which was endorsed by Government of Nepal in collaboration with development partners to reduce the burden of undernutrition in the country [23]. This plan targets to reduce undernutrition among the first thousand days lives, adolescent girls, pregnant and lactating women among the poorer groups of the community through integrated intervention. Nutritional interventions are combined with water, sanitation, hygiene, social protection, and agricultural interventions to break the strong intergenerational cycle of stunting. Similarly, in 2011, Nepal participated in a global movement called Scaling Up Nutrition, which unites national leaders, civil society, bilateral and multilateral organizations, donors, businesses, and researchers in a collective effort to improve nutrition [6]. On the other hand, based on the given current trend (i.e. 1.42% per annum), this rate of decline in stunting is not likely to move Nepal in the path to meet the WHO target of 40% fewer stunted children by 2025 [25]. However, a 3.9% annual reduction is required to achieve this global target [23]. The current prevalence of stunting (35.8%) is still very high. The challenges such as 8 % of the under five children suffering from diarrhoea and 20.9% of the rural households without toilets still remains to be tackled to minimize the prevalence [5].

Previous studies have found urban children taller than rural [24]. Of particular relevance is a study from Paciorek, Stevens et al. (2013) that analysed 141 low and middle income countries between 1985 and 2011 showing urban children are taller and heavier than their rural counterparts from the majority of countries analysed [21]. This contradicts the result of the present study. In the unadjusted analysis, the children living in the rural areas were associated with increased odds of stunting compared to their urban counterparts; however, in the adjusted analysis, no association was noted for all the survey years.

An analysis of three Cambodian Demographic Health Surveys found a significant relationship between stunting and mothers education [25], which is in accordance with the finding of this study. Rabbani, Khan et al. (2016) confirmed that mother’s education level and physical stature are statistically significant determinants for stunting [26]. Similarly, this study found that children born to mothers with primary and secondary education have lower odds of getting stunted than those who are born to mothers with no education. This may be due to higher literacy level allowing mothers to follow good practices on maternal and child health care, infant and young child feeding practices, sanitation and hygiene, which ultimately affects the nutritional status of children [27]. Incase of mothers without education, the prevalence of stunting decreased from 62 to 46% from 2001 to 2016. This reduction may be associated with the reduction in the overall proportion of mothers without education from 72 to 34% as noted in NDHS 2001 and 2016 respectively .

A large proportion of stunted children belong to the mid-western region. The mid-western development region of Nepal is the least developed region. For instance, the mid-western development region is the poorest region with the greatest difference between revenue and expenditures (− 7903.82 Nepalese Rupee) in comparison to nation’s richest region i.e. central development region generating 79.5% of the government revenue [28]. Similarly, the central region’s per capita income was $1597, which was more than the national average of $1310 and the mid-western region had the lowest per capita income of $988 [29]. The mid western development region has poor access to good health services; inadequate health facilities; a higher rate of male migration; low status of women within family groups leading to higher workload for females [9] and also lacks infrastructures such as roads, schools, hospitals, electricity, drinking water and irrigation, which has exacerbated social exclusion and increased livelihood insecurity [30]. Additionally, the inequalities in the mid-western region is associated with widespread poverty and geographical isolation [30].

The decline in prevalence of stunting among children has been similar for the three ecological regions till 2011. After 2011, it was noticed that the prevalence of stunting in the terai region declined by less than 1 % from 2011 to 2016. The low reduction in stunting in the terai region from 2011 to 2016 might be due to a larger population in terai region deprived of basic education and health related facilities [31]. In addition, this region holds ethnic populations who are socially, culturally and economically excluded from mainstream development and experience challenges to enjoy health, education and access to resources [31].

This study found that children born to poorest and poorer groups have higher odds of getting stunted than those born to richer groups. Similarly, the prevalence of stunting decreased by 18.4% from 2001 to 2016 for the poorest quintile and for the richest quintile, the reduction was by 25.6%. Additionally, the gap in prevalence of stunting between poorest and richest quintile was 25.5% in 2001, which increased to 32.7% in 2016. The reduction is not uniform among economic subgroups across the survey years, especially among lower socio-economic classes. This findings corresponds to the study done in Ghana using DHS data, which found that children belonging to the poorest households were more than twice at risk of being undernourished compared to their counterparts in the richest households [32]. It is widely accepted that when economies grow and poverty is reduced, child nutrition improves owing to greater access to food, improved maternal and child care and better public health services [33]. Alongside, instead of using blanket approach for delivering nutrition interventions, special emphasis should be given to vulnerable groups such as children belonging to poorest and poorer wealth quintile and to those born to mothers without education to balance the inequalities prevalent across different regions and subgroups by specifically bringing those behind within the reach of nutrition interventions.

The limitation of this study is that it has not given province level information on stunting, which is highly useful from policy point of view. This is because the country was divided into seven federal provinces in 2015 as per schedule 4 of the new Constitution of Nepal and there are no longer development regions in Nepal. The current system of seven provinces replaced an earlier system where Nepal was divided into five development regions. The new policies and programs in every sector including health are formulated considering the new provincial level structure. Thus, the provincial level information on stunting would highlight the current need of each province and this would help program planners and policy makers to design their interventions accordingly. However, province level data was only available for the year 2016. Hence, this study could not incorporate province level information. The causal inference between stunting and study variables is limited due to the cross sectional nature of the studies. This study doesn’t explain about caste or ethnicity, which might have influenced the inequalities in stunting. However, it has examined the relationship of stunting with mother’s education, wealth quintile etc. and have found significant association between them. The strength of this study would be that this study is based on the four large nationally representative population and large sample size warrant a high precision of the findings. Alongside, NDHS used the standardized tools, which are reliable and comparable to other developing countries.

## Conclusions

The study added to the existing knowledge that stunting have been decreasing in Nepal at national level; however, substantial inequalities have been preserved. The prevalence of stunting for children from the poorest wealth quintile is almost twice as that of children from the richest quintile, which was consistent with the results of other studies. The present study provides a scientific evidence for policy makers and programmes planners to lay down their target based on the findings.

## Abbreviations

AOR: Adjusted Odds Ratio
CI: Confidence Interval
COR: Crude Odds Ratio
DHS: Demographic Health Survey
MNSP: Multi-Sectoral Nutrition Plan
NDHS: Nepal Demographic Health Survey
PSU: Primary Sampling Unit
SD: Standard deviation
SPSS: Statistical Package for Social Science
VDC: Village development committee
WHO: World Health Organization
